# Ferromagnetism and Ru-Ru distance in SrRuO_3_ thin film grown on SrTiO_3_ (111) substrate

**DOI:** 10.1186/1556-276X-9-8

**Published:** 2014-01-07

**Authors:** Bowha Lee, O-Ung Kwon, Ran Hee Shin, William Jo, Chang Uk Jung

**Affiliations:** 1 Department of Physics, Hankuk University of Foreign Studies, Yongin 449-791, South Korea; 2 Department of Physics, Ewha Womans University, Seoul 120-750, South Korea

**Keywords:** SrRuO_3_, Ferromagnetic transition temperature, Ru-Ru nearest neighbor distance, Tolerance factor

## Abstract

Epitaxial SrRuO_3_ thin films were grown on both (100) and (111) SrTiO_3_ substrates with atomically flat surfaces that are required to grow high-quality films of materials under debate. The following notable differences were observed in the (111)-oriented SrRuO_3_ films: (1) slightly different growth mode, (2) approximately 10 K higher ferromagnetic transition temperature, and (3) better conducting behavior with higher relative resistivity ratio, than (100)_c_-oriented SrRuO_3_ films. Together with the reported results on SrRuO_3_ thin films grown on (110) SrTiO_3_ substrate, the different physical properties were discussed newly in terms of the Ru-Ru nearest neighbor distance instead of the famous tolerance factor.

**PACS:**

75.70.Ak; 75.60.Ej; 81.15.Fg

## Background

Due to its low resistivity and good chemical stability, SrRuO_3_ (SRO) is frequently used as metallic electrodes in epitaxial perovskite-heterostructure capacitors [1,2]. Film thickness, the amount of lattice mismatch, oxygen vacancy, and Ru vacancy are found to change its physical properties.

Fundamental thickness limit of itinerant ferromagnetism was observed [3]. In addition to thickness being smaller than the critical thickness (*t* < 10 unit cells), a significant amount of oxygen vacancy was also found to deteriorate its ferromagnetic properties for thicker films (*t* > > 10 unit cells). Aside from these two factors, the ferromagnetic properties of SRO, especially the ferromagnetic transition temperature, *T*_c_, have been known to be rather robust. While transport properties such as residual resistivity ratio (varying order of magnitude) are very sensitive to a tiny amount of Ru vacancy in SRO thin films grown on (100) SrTiO_3_ (STO) substrates, the ferromagnetic properties are rather immune to this tiny amount of Ru vacancy [1]. A peculiar orthorhombic-to-tetragonal structural transition with variation of the Ru-O-Ru bond angle was observed depending on the thickness and temperature of the SRO film on STO (001) substrate but this structural transition temperature was not associated with the ferromagnetic transition temperature [4].

While many previous studies have focused on (100)_c_-oriented SRO films, the in-plane magnetization of thin films on top of STO (001) substrates was smaller than out-of-plane magnetization and *T*_c_ was smaller than that of bulk SRO [5,6]. The observed small change of ferromagnetic properties in SRO films has been mostly explained simply in terms of lattice mismatch. A free-standing film made by lifting the film off its growth substrate recovered its bulk *T*_c_ and bulk saturated magnetic moment [5,6]. An SRO film having a structure most similar to the bulk SRO was made using a buffer layer and STO (110) substrate, and its magnetic anisotropy was maximum [7-9]. The observed changes in SRO films on STO (110) was explained based on the inherently lower lattice mismatch of the orthorhombic crystal along the cubic substrate’s [1-10] in-plane direction than along the cubic substrate’s [001] in-plane direction [9]. So, the lattice mismatch of orthorhombic crystal can always be smaller by choosing a cubic (110) substrate instead of a cubic (001) substrate. (In this report, we use pseudocubic notation for SRO films. (110)_orthorhombic_ is equivalent to (100)_c_ in the pseudocubic notation).

Up to now, the tolerance factor, *t* = (*r*_*A*_ + *r*_*O*_)/√2(*r*_*B*_ + *r*_*O*_), was widely regarded as the most dominant factor to determine the structural transition from cubic to lower symmetries and accompanying huge changes in magnetic and electrical properties of many perovskite oxides [10-12]. Smaller *t* < 1.0 results in a more distorted structure having a smaller Ru-O-Ru bond angle [4]. This factor is but a simple geometrical factor which cares the optimal radius of a sphere inside eight octahedra arranged at right angles and has been quite useful to explain major physical properties such as transport and magnetic properties in cubic, tetragonal, and orthorhombic colossal magnetoresistance. Recently, the structure modification effect on magnetic properties was reported in SrTi_1-*x*_Fe_*x*_O_3-*δ*_ thin films on STO (001), (110), and (111) substrates [13]. The authors tried to interpret the change of magnetostriction in terms of lattice parameter.

In this paper, we discussed the physical property changes in terms of the nearest neighbor distance of B-site ion instead of the tolerance factor. We found that STO (001) and (111) substrates are ideal to study the change of physical properties of SRO films with Ru-Ru nearest neighbor distance (Ru nn-distance) which changes in order to accommodate the Sr^2+^ ion. This is because the Ru nn-distance can be differently changed by using different surface directions of the substrates. In the rhombohedral structure of the SRO film on STO (111) substrate, the Ru nn-distance does not change much to accommodate the Sr^2+^ ion, which might be able to explain the better transport and magnetic properties in this film.

## Main text

The SRO thin films were grown on STO (001) and STO (111) substrates with a pulsed laser deposition method using a KrF excimer laser [7-9,14,15]. For simplicity, we will use ‘the SRO_100_ film’ and ‘the SRO_111_ film,’ respectively. Both substrates were initially prepared by etching and heat treatment to create step-and-terrace structures. Laser pulses of 140 to 170 mJ at 2 to 5 Hz were focused on a stoichiometric ceramic target. The substrate temperature and the oxygen partial pressure during deposition were 700°C to 760°C and approximately 100 mTorr, respectively. The thickness of the SRO film was 37 to 38 nm. We used an atomic force microscope (AFM) to check the surface morphology of the treated STO substrate and the SRO films. We performed structural analyses using a high-resolution X-ray diffractometer (HRXRD). The magnetic properties were measured with a superconducting quantum interference device (MPMS*XL*, Quantum Design, San Diego, CA, USA).

As the STO (111) surface consists of two highly polar layers of SrO_3_^4-^ and Ti^4+^, thermodynamic mixed termination is preferred to minimize the surface dipole [16]. However, atomically well-defined SrTiO_3_ (111) substrates with a strong polar interface were recently developed using a rather difficult and selective etching of SrO_3_^4-^ and thermal annealing process [12]. Chang et al. reported that simple annealing of as-polished STO (111) substrates yielded a step-and-terrace surface structure characterized by many bumps with step heights in multiples of 1/2 × *d*_111_, indicating mixed termination [16,17]. To obtain atomically flat SrTiO_3_ (111) substrates with good step-and-terrace structures with Ti termination, they reported that harsh etching is required; both an ultrasonically agitated buffered hydrofluoric acid (BHF) solution and high-temperature ultrasonic agitation of an STO (111) substrate in deionized water were needed instead of mere soaking in etchants at room temperature. The harsh etching was followed by subsequent thermal annealing in a tube furnace at 1,050°C under an O_2_ atmosphere for 1 h.

Here, we report the simple preparation of atomically well-defined SrTiO_3_ (111) substrates and subsequent growth of SRO thin films. The surface roughness, rocking curve width, and transport properties showed that the SRO film grown on the SrTiO_3_ (111) substrates was of high quality. We compared basically the growth mode, transport properties, surface morphology, and magnetic properties of these films with the SRO film grown on the SrTiO_3_ (001) substrate with different structure deformation. Due to the additional danger accompanying the use of the ultrasonic agitator with BHF, we etched the STO (111) substrate using two different soaking times at room temperature, followed by annealing the etched substrate in a tube furnace at approximately 1,000°C under an O_2_ atmosphere for approximately 5 h. (For the STO (001) substrate, the typical soaking time was 15 to 30 s.) We found that simply increasing the BHF soak time worked very well for the STO (111) substrate without resorting to a more complicated method [17,18]. (Connell et al. found that atomically flat STO (001) substrate can be prepared even without the use of dangerous BHF [19]).

## Discussion

**Figure 1:**
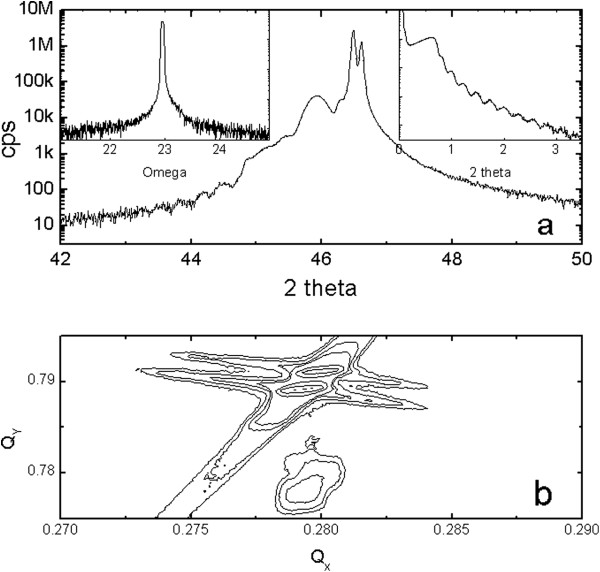
**HRXRD results for the SrRuO**_**3**_**/SrTiO**_**3 **_**(001) substrate. (a)** XRD *θ* to 2*θ* scan patterns. The left inset shows the rocking curve of the SrRuO_3_ (200)_c_ peak. FWHM was as small as 0.057°. The right inset shows good oscillations at low angles due to the uniform thickness of about 38 nm. **(b)** X-ray reciprocal space mapping around the STO (114) plane showed well-developed peaks for SrRuO_3_ in the lower region and two strong substrate peaks in the upper region.

Figure 1 shows HRXRD results for the SRO_100_ film. There was a strong SRO film peak on the left side of two large substrate peaks near 2*θ* = 46.46°. (The two strongest and well-separated substrate peaks corresponded to Cu *K*α_1_ and *K*α_2_ sources in the X-ray tube.) The calculated lattice constant of the SRO, *d*_200c_ = 1.975 Å = 3.950 Å/2, indicated a high-quality film^a^[20,21]. Oxygen vacancies usually induce lattice expansion resulting in a much larger 2 × *d*_200c_ than 3.950 Å. The high crystallinity was also confirmed by the value of the full width at half maximum (FWHM) rocking curve of the SRO (200)_c_ peak. The value was as small as 0.057°, which is consistent with the value of 0.06° reported previously [22]. The right inset of Figure 1 shows good oscillations at low angles due to the uniform thickness (*t* ~ 38 nm) of the SRO_100_ film. X-ray reciprocal space mapping around the STO (114) plane in Figure 1b showed well-developed peaks for SrRuO_3_ in the lower region and two strong substrate peaks in the upper region. The strong peaks for SRO were well centered and the obtained *d*_400c_ was consistent with the value of *d*_200c_ in the *θ* to 2*θ* scan. The position of the film peak along the horizontal *Q*_*x*_ axis was the same as that of the substrate peak, indicating that the SRO_100_ film was grown coherently on the STO (001) substrate, with the same in-plane lattice constant. This indicated that the SRO_100_ film was under compressive strain.

**Figure 2:**
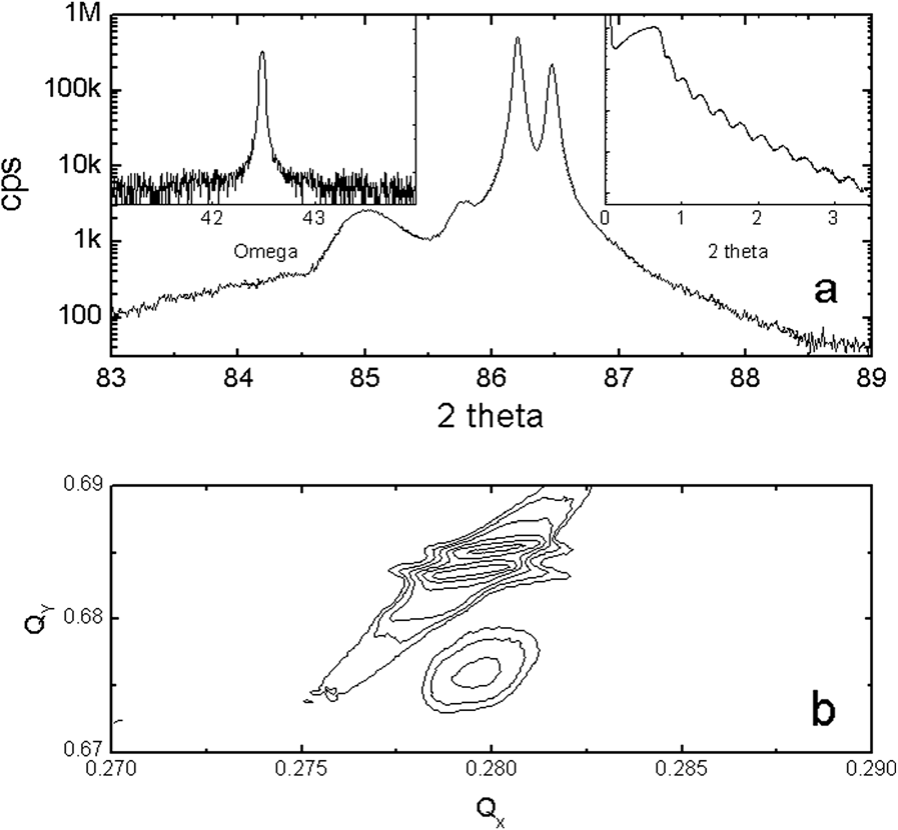
**HRXRD results for the SrRuO**_**3**_**/SrTiO**_**3 **_**(111) substrate. (a)** XRD *θ* to 2*θ* scan patterns. The left inset shows the rocking curve of the SrRuO_3_ (222) peak. FWHM was as small as 0.052°. The right inset shows good oscillations at low angles due to the uniform thickness of about 38 nm. **(b)** X-ray reciprocal space mapping around the STO (312) plane showed well-developed peaks for SrRuO_3_ in the lower region and two strong substrate peaks in the upper region.

Figure 2 shows HRXRD results for the SRO_111_ film. There was a strong SRO film peak near 2*θ* = 85.03° together with the strongest substrate peak near 2*θ* = 86.21°. (The peak near 2*θ* = 85.80° was not due to impurities but to spurious light from the X-ray source.) The calculated lattice constant of the SRO was *d*_222_ = 1.140 Å = 3.949 Å/2√3, again indicating a high-quality film. The high crystallinity of the SRO_111_ film was also confirmed by the value of the full width at half maximum of the SRO (222) peak. This value was as small as 0.052°, smaller than that of the SRO_100_ film. The right inset of Figure 2 shows good oscillations at low angles due to the uniform thickness of about 37 nm. X-ray reciprocal space mapping around the STO (312) plane shown in Figure 2b contains well-developed peaks for the SRO_111_ film in the lower region and two strong substrate peaks in the upper region. The strong peaks for SRO were well centered and the obtained *d*_111_ was consistent with the *d*_222_ obtained in the *θ* to 2*θ* scan. The position of the film peak along the horizontal *Q*_*x*_ axis was the same as that of the substrate peak, indicating that the SRO_111_ film was grown coherently on the STO (111) substrate, with the same in-plane lattice constant. This indicated that the SRO_111_ film was under compressive strain. When we compared the HRXRD data of the two films, we found that the unit cell volume of the SRO_111_ film was nearly equal to that of the SRO_100_ film (*V*_pseudocubic_ = 3.905^2^ × 3.949 Å^3^) and with comparable thicknesses.

**Figure 3:**
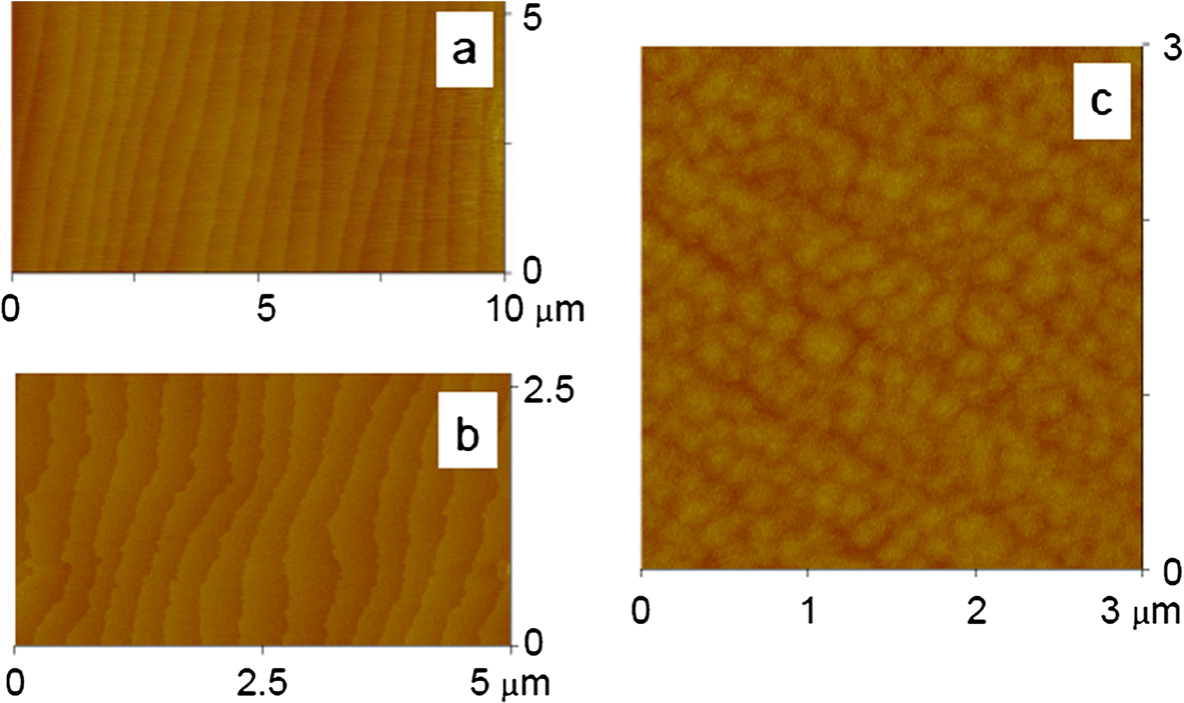
**Surface images taken with an atomic force microscope. (a)** SrTiO_3_ (111) substrate prepared by etching and subsequent annealing, **(b)** SrRuO_3_/SrTiO_3_ (001), and **(c)** SrRuO_3_/SrTiO_3_ (111).

We used AFM to observe the surface of the STO (111) substrate, which was used for the growth of the SRO thin film, as shown in Figure 3a. A step-and-terrace structure comparable to that reported previously by harsh etching could be clearly seen [17]. Figures 3b,c shows the surface morphologies of the SRO_100_ film and the SRO_111_ film, respectively. The SRO_100_ film had the well-known step-and-terrace structure consistent with its step-flow growth mode, but the SRO_111_ film showed a rather different surface morphology. With a background of step-and-terrace, there appeared many small islands within a height of one unit cell. The existence of the islands indicated a different growth mode from the step-flow growth mode typically observed in high-quality SRO films grown on STO (001) substrates. While there was a model that attempted to rationalize the diverse growth modes observed in pulsed laser deposition of SRO on SrTiO_3_ (001) substrates, the existence of a highly polar surface of a Ti^4+^-terminated STO (111) surface may be another factor to avoid step flow mode [23,24]. The RMS roughness measured was 0.25 nm, which was much smaller than the value of 0.6 to 4.0 nm reported previously^b^[22].

**Figure 4:**
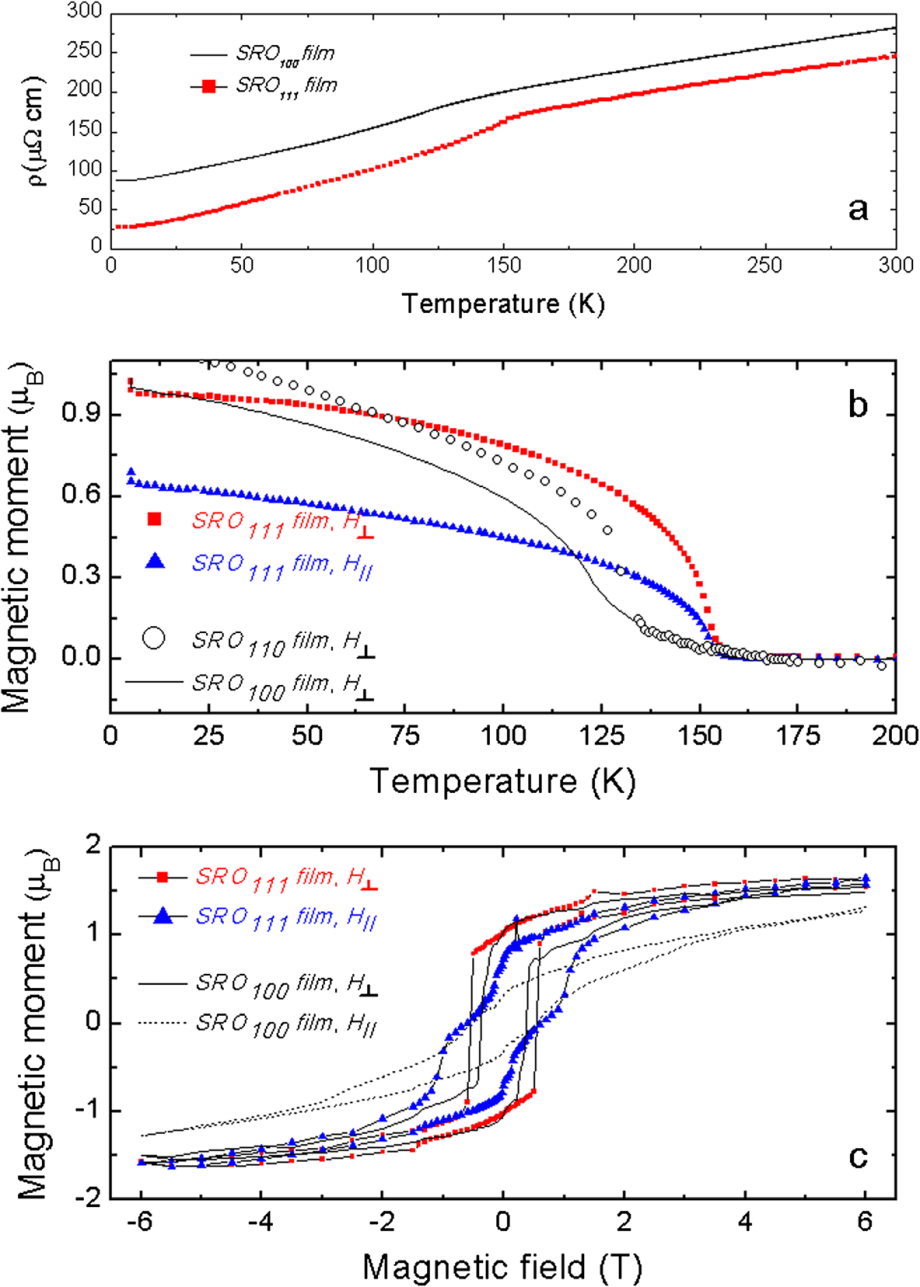
**Transport and magnetic properties of SrRuO**_**3**_**/SrTiO**_**3 **_**(001) and SrRuO**_**3**_**/SrTiO**_**3 **_**(111).** For SrRuO_3_/SrTiO_3_ (111), magnetization was measured in two field directions with respect to the substrate: surface normal and in-plane directions. **(a)** Resistivity curves. **(b)** Magnetization curves together with those of SrRuO_3_ films on SrTiO_3_ (001) and STO (110) substrates reported by Jung et al. [7]. **(c)** Magnetic hysteresis curves at 5 K.

Figure 4a shows the temperature dependence of the resistivity of the two films. For the SRO_100_ film, the room temperature resistivity was *ρ*(300 K) ~ 280 μΩ · cm and the resistivity at 4 K was approximately 87 μΩ · cm with a residual resistivity ratio (RRR) of 3.2. While the resistivity at low temperatures was higher than expected, the upturn of resistivity at low temperatures observed for other group's SRO films was not observed in our SRO_100_ film [25]. The kink in the resistivity near 150 K is known to be caused by the ferromagnetic transition temperature. All these features are consistent with those reported by other groups [5,6]. The resistivity of the SRO_111_ film showed three different features in comparison to that of the SRO_100_ film. First, the location of the resistivity kink on the temperature axis was also shifted to a higher temperature, implying a high ferromagnetic transition temperature. Second, the overall resistivity value for the SRO_111_ film was smaller than that for the SRO_100_ film, especially at low temperatures. Finally, the RRR (approximately 9) is higher.

There are many reasons that affect the different RRR values in epitaxially grown SrRuO_3_ thin films. Chemical doping like (Ca,Sr)RuO_3_ or epitaxial strain caused by using different substrates can change the bandwidth (thus transport properties) probably due to different Ru-O-Ru bond angles [1]. If we use the same substrate for thin film growth, there are other factors that affect RRR. Oxygen vacancy and/or Ru vacancy can cause low RRR values and these accompany with expansion of the lattice. For example, a recent review paper correlated the unit cell volume with RRR [1]. According to the review paper, an SRO with an orthorhombic unit cell volume of 240.9 Å^3^ ((=3.905^2^ × 3.950 × 4) should have RRR ~ 20. However, in our case, RRRs were 3 and 9 for the SRO_100_ film and the SRO_111_ film, respectively. A single-crystalline SRO thin film on STO (110) substrate having an orthorhombic unit cell volume of 240.9 Å^3^ was reported to have RRR ~ 8 [26]. So, a simple explanation in terms of structural factor such as volume expansion is not enough to explain the different RRR values even though we accept that PLD-grown SRO films have more tendency to have larger lattice volumes and have lower RRR values.

Siemons et al. estimated that the Ru vacancy concentration causing drastic change of RRR is much smaller than a few percent for the range of samples they studied, from the fact that the decrease of the Curie temperature is as small as approximately 10 K [27]. Thus, the effect of a very small amount of Ru vacancy in SRO thin films seems to be critical for RRR but should be much smaller than the effect of strain on the ferromagnetic properties [27]. This is consistent with the observation of robust low-spin configuration for nearly all thin films of SrRuO_3_.

Figure 4b shows the temperature dependence of the magnetization at 500 Oe after high field cooling at 7 T. [The same specimen was used for these measurements by only changing the field direction with respect to the crystallographic axis - one along the in-plane direction, *H*_//_and the other along the surface normal direction, *H*_⟂_.] For the SRO_111_ film, the magnitude of magnetization along the surface normal direction was larger than that along the in-plane direction. This was similar to the observations for the SRO_100_ film and was interpreted in terms of compressive strain [5,6]. To estimate the changes in the ferromagnetic transition temperature, we plotted magnetization of the SRO_100_ film and the SRO film grown on STO (110) substrate on the same plot [7]. From Fig. 4(b), it can be seen that the ferromagnetic transition temperature of the SRO_111_ film is about 10 K higher than those of the SRO_100_ film and SRO film grown on STO (110) substrate. These increased ferromagnetic transition temperatures of films grown on a cubic (111) substrate were also reported for manganese oxide [28-30].

Figure 4c shows magnetic hysteresis curves at 5 K for applied fields along two directions. Here, we found that magnetization along the surface normal direction increased more rapidly than that along the in-plane direction. For fields along the surface normal direction, the coercive field was very well defined for both films. The coercive field for the SRO_111_ film was approximately 0.7 T, which was slightly larger than the value of approximately 0.5 T for the SRO_100_ film. Finally, we found that the saturated magnetic moments with a 6-T applied field were smaller than 2 μ_B_/Ru. This was in contrast to the observed approximately 3.5 μ_B_/Ru in the SRO film grown on STO (111) substrate [22]. Note that we grew a very high-quality film on top of the STO (111) substrates with step-and-terrace structures.

Similarly, it has not been reported that volume change due to a small amount of Ru vacancy causing subtle change of the Ru-O-Ru bond angle can induce a significant change of spin configuration in SRO [1,26]. The orthorhombic-to-tetragonal structural transition temperature *T*_OT_ as a function of the SRO film thickness did not show a correlation with the ferromagnetic transition temperature [31].

Previously, the difference of RRR and *T*_c_ has been explained by oxygen vacancy, Ru vacancy, and surface difference. However, the SRO_100_ film and the SRO_111_ film have nearly the same lattice parameters and unit cell volumes because the volume difference between the two films is within the error bar of HRXRD. So, the vacancies could not explain the different RRR and *T*_c_ between the two films. Since the films are as thick as approximately 100 unit cells, which is enough to neglect surface dependence, surface effects on its physical properties must be excluded.

**Figure 5:**
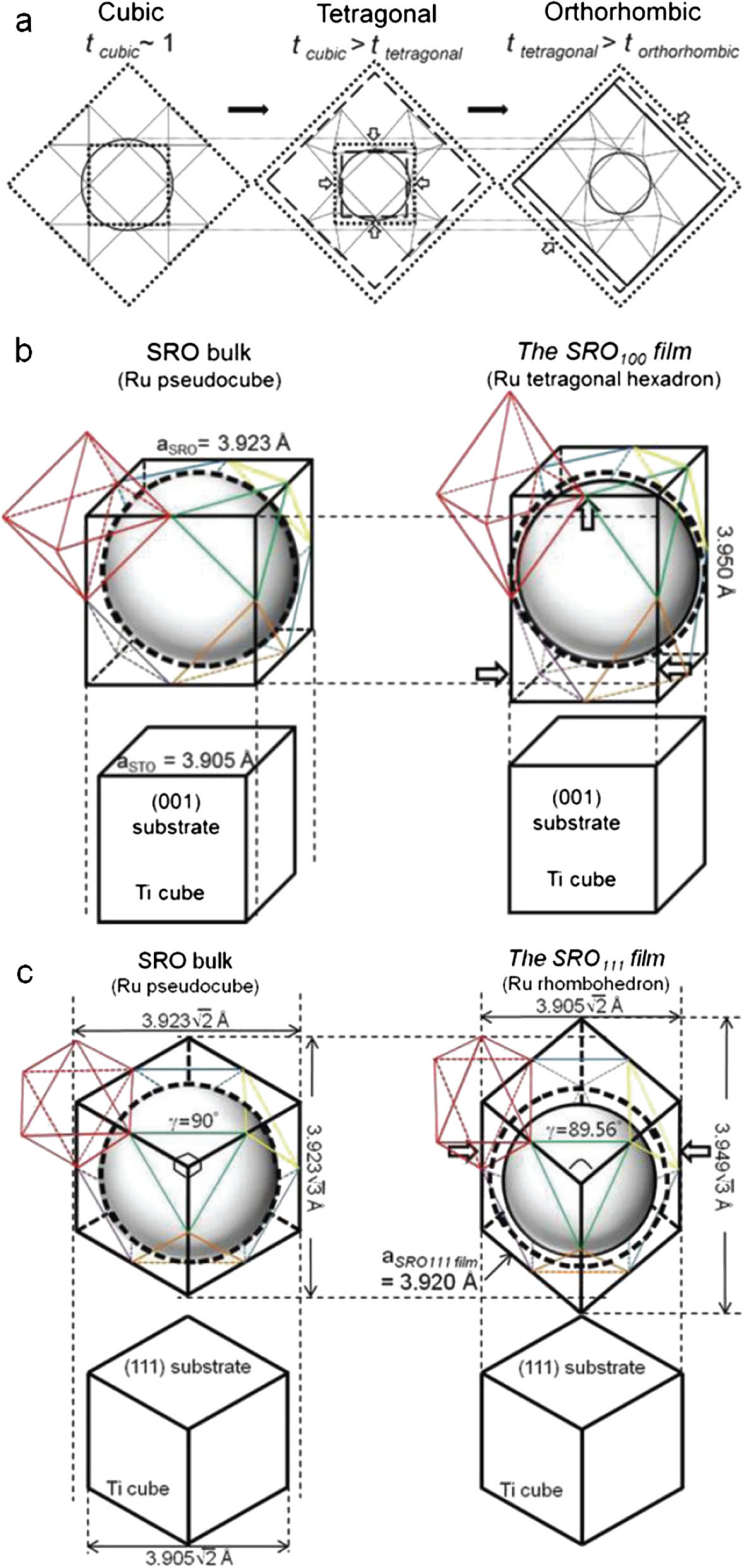
**Schematic diagram of structural change in terms of octahedral distortion, hollow inscribed sphere, and its surrounding eight octahedra. (a)** Perovskite oxide as the tolerance factor decreases from approximately 1, **(b)** the SRO_100_ film, and **(c)** the SRO_111_ film with bulk SRO. The Ru nn-distance in the film depended critically on the type of substrate orientation.

Figure 5a shows the structural change of perovskite oxide as the tolerance factor decreases from 1.0. As *t* = (*r*_A_ + *r*_O_)/√2(*r*_B_ + *r*_O_) decreases due to the insufficient radius of the A site ion inside the cube consisting of eight BO_6_ octahedra, the octahedra rotate and tilt to prepare more suitable (smaller) space for smaller A site ions. The tolerance factor has a direct relation with the B-O-B buckling angle and thus electron transfer interaction between *d* electron in the B site and O 2*p* states. Thus, the tolerance factor in the perovskite was the most dominant factor to determine electric and/or magnetic properties in most manganese oxides and nickelates [10-12].

Figure 5b,c shows the different effects of strain on the nearest neighbor distance between the adjacent Ru ions (≡Ru nn-distance) depending on the substrate surface orientation. The lattice of the SRO_100_ film is simply elongated along the *c*-axis direction while those along the two in-plane lattices shrank. The result is that the Ru nn-distance along the *c*-axis becomes larger than that of the bulk SRO (3.950 Å > 3.923 Å, approximately 0.69%) and that along two in-plane axes becomes smaller (3.905 Å < 3.923 Å, approximately -0.46%) due to the coherent growth through the epitaxial strain.

If we grow SRO on top of STO (111) substrate, SRO will receive compressive strain. The deformation of SRO occurs in the following way: A Ru pseudocube of SRO consisting of eight Ru ions at each corner will transform to a rhombohedron. By using the in-plane lattice constant and out-of plane lattice constant data in Figure 2, we could get the shape of the rhombus of the rhombohedron: side length of approximately 3.920 Å and angle of approximately 89.56°. In summary, through the rhombohedral distortion, the Ru nn-distance does change very little (approximately 0.003 Å) from its bulk value of 3.923 Å by reducing the Ru-Ru-Ru angle *γ* from 90° to only approximately 0.44°. Another point is that the ‘Ru cube’ could hold ions larger than the Sr ion at its center since Ru is larger than Ti. (SrTiO_3_ is cubic. The ‘Ti cube’ has a lattice constant of 3.905 Å.) Thus, the bulk SRO structure was made by decreasing the inner hollow space of the cube by having a buckling angle and thus has an orthorhombic structure. In the SRO_111_ film, the Ru cube changed to a rhombohedron and its inner hollow volume is closer to the optimum value to have the Sr ion at its center which is a little bit smaller to fill the inner space of the undistorted Ru cube having a lattice constant of approximately 3.923 Å^c^.

When the SRO film is grown with different strain directions, there are three categories that we might consider as key parameters: (1) Ru-O distance, (2) Ru-O-Ru buckling angle, (3) Ru nn-distance. Previous reports have mainly focused on Ru-O distance and Ru-O-Ru buckling angle, which are in the scheme of the tolerance factor. However, the tolerance factor mostly covers cubic, tetragonal, and orthorhombic structures. In the SRO_111_ film, we could keep nearly the bulk SRO value of the Ru nn-distance more easily while the Ru nn-distance of the SRO_100_ film was quite reduced along the in-plane direction. The ability of keeping the Ru nn-distance closer to the bulk value seems to be one of the main factors to obtain higher RRR and *T*_c_ in the SRO_111_ film compared to the SRO_100_ film. This scenario can be generalized to other cases. The smaller lattice mismatch in SRO/STO (110) compared to SRO/STO (001) means the a smaller disturbance to the original Ru nn-distance [7,9]. With *d*_1-10_ = 3.905 Å/√2 and *d*_110_ = 3.905 Å/√2, the Ru nn-distance and Ru-Ru-Ru angle are approximately 3.928 Å and approximately 89.34° along the rhombus side and 3.905 Å and 90° along the rectangular side of SRO (110) film, repectively [7-9]. In summary, the major change of Ru nn-distance from the pseudocubic bulk SRO value of 3.923 Å is approximately -0.018 Å for the SRO (100) film, approximately -0.006 Å and approximately -0.017 Å for the SRO (110) film, and approximately -0.003 Å for the SRO (111) film. Thus, the nearest neighbor distance between B-site ions seems to be as good as the tolerance factor in perovskite thin films and even better if the strain pushes lower symmetry like in rhombohedral structures.

## Conclusions

We made high-quality SrRuO_3_ thin films on SrTiO_3_ (111) and SrTiO_3_ (001) substrates with atomically flat surfaces. The SrRuO_3_ thin films on SrTiO_3_ (111) substrate showed (1) a slightly different growth mode, (2) approximately 10 K higher ferromagnetic transition temperature, and (3) better conducting behavior with a higher relative resistivity ratio, than (100)_c_-oriented SrRuO_3_ films. The oxygen and Ru vacancies are not dominant factors for the difference because of the same unit cell volume for both films. The differences in the magnetic and electrical properties should be interpreted in terms of other factors, probably different structural deformation of the SrRuO_3_ unit cell. In the SRO_111_ film, we could nearly keep the bulk SRO value of the Ru nn-distance more easily while the Ru nn-distances of the SRO_100_ film and of the SRO_110_ film were quite changed along the in-plane direction. We propose Ru nearest neighbor distance as a new concept, for explaining strain effects in perovskite oxide thin films grown on different surfaces of cubic substrates. Finally, (111)_c_-oriented SrRuO_3_ films revealed no signatures of high-spin states of Ru.

## Endnotes

^a^Recent studies on the detailed crystal structure of SRO thin films showed that the crystal structure of the film depended on the thickness, temperature, and type of in-plane strain. A thicker SRO film on a SrTiO_3_ (001) substrate has a very slight distortion from tetragonal to monoclinic at room temperature.

^b^We found that the optimal growth conditions for the SRO_111_ film in terms of surface morphology were much narrower than those for the SRO_100_ film.

^c^The ideal Ru cube should have a lattice constant larger than 3.923 Å. One may have to make Ba_*x*_Sr_1-*x*_RuO_3_ in cubic phase and measure its lattice constant.

## Competing interests

The authors declare that they have no competing interests.

## Authors’ contributions

O-UK and RHS made Figure 5, found good references, and contributed to the introduction of the key concept. CUJ managed the whole experimental results and organized the manuscript as the corresponding author. BL and WJ joined the discussion. All authors read and approved the final manuscript.
